# Teenage Reproductive Health: Pregnancy, Contraception, Unsafe Abortion, Fertility

**DOI:** 10.3390/ijerph15061176

**Published:** 2018-06-05

**Authors:** Jon Øyvind Odland

**Affiliations:** Department of Public Health and Nursing, Faculty of Health Sciences, Norwegian University of Science and Technology (NTNU), 7491 Trondheim, Norway; jon.o.odland@ntnu.no

We are proud to present 14 papers with focus on teenager health in this Special Issue entitled “Teenage Reproductive Health: Pregnancy, Contraception, Unsafe Abortion, Fertility”.

Maternal mortality is still globally high and reducing it is a top priority. Teenage pregnancies have more complications and are also unwanted in many cases. This contributes to the high maternal mortality with both obstetric complications and burden of unsafe abortion. Additionally, many teenagers live in areas with heavy pollution that affects the mother and the unborn child. Global public health is a very important issue that aims to prevent disease, prolong life, and promote physical, mental and social well-being. Teenagers are the future, and maternal death is a disaster that should be prevented. Hence, research should aim to improve teenage reproductive health and influence policy makers. There are a variety of topics in this issue, with some conclusions and ways forward as described in different papers.

Oppong-Darko et al. [1] describe the dilemma between religious beliefs and traditions and the conflict with reproductive health and abortion laws in Ghana, which is a typical low-income country (LMIC). They conclude that the midwives make it clear that unsafe abortions are common, stigmatizing and contributing to maternal mortality, which are issues that must be addressed. They introduce various suggestions to reduce this preventable tragedy.

Grossman et al. [2] have a very interesting paper about parents’ conversations with their teenagers about reproductive health and risks in sexual behavior. It seems like once teens entered high school, more parents described feelings comfortable with their conversations. However, parents also more often reported that their teens responded more negatively to the communication in high school than they had in middle school. These findings may help parents to anticipate their own as well as their teens’ responses to family conversations about sex at different developmental time points and to strategize how to effectively talk with their teens about sex and relationships to improve their teens’ overall reproductive health.

von Rosen et al. [3] describe how sexually transmitted infections (STIs) pose a significant threat to individual and public health. They disproportionately affect adolescents and young adults. In a cross-sectional study, they assessed self-rated and factual STI knowledge in a sample of 9th graders in 13 secondary schools in Berlin, Germany. Differences by age, gender, migrant background and school type were quantified. Knowledge of human immunodeficiency virus (HIV) was widespread, but other STIs were less known. Almost half of the participants had never heard of chlamydia, 10.8% knew the HPV vaccination, and only 2.2% were aware that no cure exists for HPV infection. While boys were more likely to describe their knowledge as good, there was no general gender superiority in factual knowledge. Overall, children of immigrants and students in the least academic schools had less knowledge. It seems that adolescents suffer from suboptimal levels of knowledge on STIs beyond HIV. Urgent efforts are needed to improve adolescent STI knowledge in order to improve the uptake of primary and secondary prevention.

Ninsiima et al. [4] went deep into the gender aspect in developing countries with their report from Uganda and wrote a paper titled “Girls Have More Challenges; They Need to Be Locked Up”: A Qualitative Study of Gender Norms and the Sexuality of Young Adolescents in Uganda. Unequal power and gender norms expose adolescent girls to high risks of HIV, early marriages, pregnancies and coerced sex. In Uganda, almost half of the girls below the age of 18 are already married or pregnant, which poses a danger to the lives of young girls. This study explores, in a very good way, the social construction of gender norms from early childhood, and how it influences adolescents’ agency. Adolescents’ agency appears constrained by context-specific obstacles. Programs targeting behavioral change need to begin early in the lives of young children. They should target teachers and parents about the values of gender equality and strengthen the legal system to create an enabling environment to address the health and wellbeing of adolescents.

Pradhan et al. [5] published a paper titled Factors Associated with Pregnancy among Married Adolescents in Nepal: Secondary Analysis of the National Demographic and Health Surveys from 2001 to 2011. They still find higher risk associated with living in the least resourced region, early sexual debut, and older husband. Despite national efforts to reduce pregnancies among married adolescent women in Nepal, prevalence remains high. Integrated and cross-sectoral prevention efforts are urgently required. A reduction in poverty and an improvement in infrastructure may also lead to lower rates of adolescent pregnancy.

Contributions to international health science from Russian authors are rapidly increasing. Usynina et al. [6] discuss adverse pregnancy outcomes among adolescents in Northwest Russia. The study is based on the upcoming and rapidly developing Arkhangelsk Birth Registry, aiming to assess whether adolescents have an increased risk of adverse pregnancy outcomes (APO), compared to adult women. Adolescents were more likely to be underweight, smoke, have infections of the kidney and the genital tract, compared to adult women. Compared to adults, adolescents were at lower risk of low birth weight, a 5 min Apgar score <7, and need for neonatal transfer. Adolescents had no increased risk of other APO studied in the adjusted analysis, suggesting that a constellation of other factors, but not young age per se, is associated with APO in the study setting.

Lafontan el al. state that it is very simple and easy to use electronic baby monitoring in Tanzania [7]. They carried out semi-structured individual interviews post-labor at two hospitals in Tanzania. The results indicated that the use of the monitor positively affected the women’s birth experience. It provided much-needed reassurance about the wellbeing of the child. The women considered that wearing the Moyo improved care due to an increase in communication and attention from birth attendants. However, the women did not fully understand the purpose and function of the device and overestimated its capabilities.

Scientific literature from Kazakhstan is increasing and improving. The paper from Dauletyarova et al. [8] discusses if Kazakhstani women are satisfied with antenatal care, implementing the WHO tool to assess the quality of antenatal services. Ninety percent of the women were satisfied with the antenatal care. Women who were dissatisfied had lower education. These women would have preferred more checkups, shorter intervals between checkups, more time with care providers and shorter waiting times. The overall dissatisfaction was associated with long waiting time and insufficient information on general health in pregnancy, results of laboratory tests, treatment during pregnancy and breastfeeding.

Abortion policies are very important in teenager health in all parts of the world. Frederico et al. [9] discuss factors influencing abortion decision-making of young women in Mozambique. The study found determinants at different levels, including the low degree of autonomy for women, the limited availability of health facilities providing abortion services and a lack of patient-centeredness of health services. Strategies are suggested to increase knowledge of abortion rights and services, and to improve the quality and accessibility of abortion services in Mozambique.

Tsikouras et al. [10] describe ten years of experience in contraception options for teenagers in a family planning center in Greece, comparing its situation with developing countries. They conclude that during adolescence, the existence of a family planning center and participation in family planning programs play a crucial role in helping the teenagers to improve their knowledge and choose an effective contraception method. This is a very important and general finding.

Back in Africa, Odland et al. [11] discuss a serious reduction of the use of manual vacuum aspiration (MVA) in hospitals in Malawi. It is a cheap and safe method, included in national strategies, but with variable use in daily routines. However, there was a major increase in MVA application at one district hospital, probably because of good education and dedicated leadership. Even with national guidelines, the implementation and follow up is highly depending on local engagement and leadership.

Kemigisha et al. [12] deliver a very interesting report on adolescents’ sexual wellbeing in Southwestern Uganda. The objective of the study was to assess sexual wellbeing in a broad sense (i.e., body image, self-esteem, and gender equitable norms) and associated factors in young adolescents in Uganda. The study with 58% females was carried out in 2016. Self-esteem and body image scores were high in both genders, but girls had higher scores, compared to boys for all outcomes. A higher age and being sexually active were associated with lower scores on gender equitable norms. Gender equitable norms scores decreased with increased age of adolescents. Comprehensive and timely sexuality education programs focusing on gender differences and norms were recommended.

Clarke et al. [13] have the only US study in the Special Issue. They discuss the histories of maltreated adolescents in treatment programs in Oregon, through a qualitative study. The results highlight the need for providing adolescent girls with reliable and practical information about risky sexual behavior and drug use from reliable and trustworthy helping professionals. Strategies for developing and maintaining trust and delivering specific content are important in all programs and their implementation.

Finally, Sharma and Nam [14] describe the condom use at last sexual intercourse and its correlates among males and females aged 15–49 Years in Nepal. Being unmarried was the most important predictor of condom use among males. Higher education was associated with increased likelihood of condom use in females. However, mobility, having multiple sexual partners and HIV knowledge were not significant correlates of condom use in both sexes. A big difference was observed in the variance accounted for males and females; indicating use of condoms was poorly predicted by the variables included in the study among females. Condom use was more associated with sociodemographic factors than with sexual behaviors and HIV knowledge.

There is a very important public health aspect in all the published papers in this Special Issue. Another good development is that young scientists are using the *International Journal of Environmental Research and Public Health* as a starting point in their career. All papers have been through a thorough review process, and many of them are now basis for the study towards academic degrees and introduction to further studies. Due to the age connection, young scientists are writing about health issues for young people. Let us hope for a new Special Issue soon, with contributions from other parts of the world. Teenagers have special health concerns in a very important transition period from child to adult. This is independent of geography, culture and religion. In total, this Special Issue provides very useful information for all parts of the world, even for those who are not presented in this nice collection of papers.
